# Correlation between National Influenza Surveillance Data and Search Queries from Mobile Devices and Desktops in South Korea

**DOI:** 10.1371/journal.pone.0158539

**Published:** 2016-07-08

**Authors:** Soo-Yong Shin, Taerim Kim, Dong-Woo Seo, Chang Hwan Sohn, Sung-Hoon Kim, Seung Mok Ryoo, Yoon-Seon Lee, Jae Ho Lee, Won Young Kim, Kyoung Soo Lim

**Affiliations:** 1 Department of Biomedical Informatics, Asan Medical Center, Seoul 138–736, Korea; 2 Department of Emergency Medicine, University of Ulsan, College of Medicine, Asan Medical Center, Seoul 138–736, Korea; 3 Department of Anesthesiology and Pain Medicine, University of Ulsan, College of Medicine, Asan Medical Center, Seoul 138–736, Korea; New York City Department of Health and Mental Hygiene, UNITED STATES

## Abstract

**Background:**

Digital surveillance using internet search queries can improve both the sensitivity and timeliness of the detection of a health event, such as an influenza outbreak. While it has recently been estimated that the mobile search volume surpasses the desktop search volume and mobile search patterns differ from desktop search patterns, the previous digital surveillance systems did not distinguish mobile and desktop search queries. The purpose of this study was to compare the performance of mobile and desktop search queries in terms of digital influenza surveillance.

**Methods and Results:**

The study period was from September 6, 2010 through August 30, 2014, which consisted of four epidemiological years. Influenza-like illness (ILI) and virologic surveillance data from the Korea Centers for Disease Control and Prevention were used. A total of 210 combined queries from our previous survey work were used for this study. Mobile and desktop weekly search data were extracted from Naver, which is the largest search engine in Korea. Spearman’s correlation analysis was used to examine the correlation of the mobile and desktop data with ILI and virologic data in Korea. We also performed lag correlation analysis. We observed that the influenza surveillance performance of mobile search queries matched or exceeded that of desktop search queries over time. The mean correlation coefficients of mobile search queries and the number of queries with an r-value of ≥ 0.7 equaled or became greater than those of desktop searches over the four epidemiological years. A lag correlation analysis of up to two weeks showed similar trends.

**Conclusion:**

Our study shows that mobile search queries for influenza surveillance have equaled or even become greater than desktop search queries over time. In the future development of influenza surveillance using search queries, the recognition of changing trend of mobile search data could be necessary.

## Introduction

Syndromic surveillance is defined as a dynamic process of collecting near real-time data on symptom clusters that are suggestive of a biological disease outbreak [1, 2]. With international concerns about emerging infectious diseases, bioterrorism, and pandemics that threaten human or veterinary public health, the importance of syndromic surveillance systems has increased [3–6]. For example, the 2009 H1N1 influenza pandemic highlighted the need for a syndromic surveillance system to inform policy and plan for effective health system responses [4].

Conventional surveillance systems depend on case reporting to report disease activity from sentinel clinics and laboratories. For example, influenza surveillance is recommended to monitor influenza-like illness (ILI) reports and influenza virus infections [5]. Because time delays in case reporting and confirmation can interfere with the early detection of outbreaks or increases in influenza cases in the community [5], digital surveillance could improve both the sensitivity and timeliness of health event detection [5, 7].

Recently, based on the rapid progress of information technology, online resources such as search queries [8–11], Twitter [12–14], Wikipedia access [15, 16], Google AdSense [17], and homepages [18, 19] have been highlighted as promising data sources for influenza surveillance. Of these, internet search queries have been widely used to predict influenza outbreaks. Google Flu Trends, which has recently ended their service, has shown a high correlation with conventional ILI surveillance data [9]. We also developed a surveillance model for Korea using query data from Google [10] and the Korean search engine, Daum [11]. Our previous work showed that the national influenza surveillance data from the Korea Centers for Disease Control and Prevention (KCDC) were highly correlated with search queries in Google and Daum [10, 11]. However, there are some criticisms of digital surveillance, such as Google Flu Trends [20–22]. Digital surveillance cannot be a substitute for conventional disease surveillance systems [10, 20, 22, 23]. In addition, there are the problems of noise consisting of changing user behavior, media-stoked panic, and changes in search engine algorithms [11, 20, 23].

It has recently been estimated that the mobile search volume has surpassed that of desktop searches because of the wide adoption of mobile devices [24–26] and mobile search patterns differ from desktop search patterns [27–32]. However, despite the fact that the volume of mobile searches is rapidly expanding, there is little evidence for the impact of these changes on digital surveillance performance. Previous studies on digital surveillance systems did not distinguish mobile and desktop search queries. The purpose of this study was to compare the performance of mobile and desktop search queries in terms of digital influenza surveillance.

## Methods

### Study period

The study period ran from September 6, 2010 (week 36) through August 30, 2014 (week 35), which consisted of four epidemiological years (2010/11, 2011/12, 2012/13, 2013/14). Analyses were performed by epidemiological year, defined as the period from week 36 through week 35 of the subsequent year by KCDC [33]. The first day of any epidemiological week is Sunday. Week numbering is sequential, beginning with one, and week one of a Korean epidemiological year is the first week of the year that includes January 1 [33].

### Data collection

Mobile and desktop weekly search queries were extracted from Naver Trends, which is freely available [34]. Naver was chosen since it is the largest search engine in Korea, with almost 80% of the Korean search market [35] and other search engines do not distinguish mobile/desktop trends. We downloaded weekly search query data from Naver Trends in comma separated value (CSV) format. This data was assigned a value between 0 and 100 by dividing the number of each combined query by the total number of search queries for a specific period (weekly in this study). Naver did not provide the absolute number of query searches like the other search engine companies. Naver provided this data separately for mobile and desktop, but the exact algorithm was unknown. We provided raw data in the supporting information (S1 File).

KCDC ILI is defined as a fever of 38°C with a cough and/or a sore throat. ILI surveillance consists of 850 sentinel clinics across the nation [33]. These clinics report the weekly percentage of outpatients who meet the case definition of ILI. The virologic surveillance data are weekly laboratory tests showing the positive rates for the influenza virus. This network consists of 91 laboratories across the nation. We downloaded the publicly available data from the KCDC website for the same study period [33]. These data were provided in the “Weekly Sentinel Surveillance Report” document [33], and we manually extracted ILI and virologic data from these documents. In our previous work [11], we performed a survey to gather population search queries related to influenza and we combined the queries from the survey results to reflect people’s search behavior[27]. The resulting queries do not include identifying patient information and are publicly available [11]. A total of 210 combined queries were used for this study [11] (S1 File). All queries with significant values were included in this study regardless of whether they were from desktop or mobile searches.

### Statistical analysis

Spearman’s correlation analysis was used to examine the correlation of the mobile and desktop data from Naver Trends with ILI and virologic data from KCDC. We used IBM SPSS Statistics software, version 20 (IBM Corporation, Armonk, NY). Strong correlation was defined as a correlation coefficient r-value of ≥ 0.7. To assess temporal relationships between Naver Trends and KCDC data for up to two weeks, we performed lag correlation analysis. The creators of disease surveillance models, such as Google Flu Trends, have claimed that the estimations of their models are 1–2 weeks ahead of the reports published by the government [9]. Given the difference in the performance between models for disease surveillance and individual internet time series, we thought that the two-week lag analysis was sufficient. Significance was set at *P* < 0.05.

## Results

### Correlation between the KCDC ILI data and search queries

**Fig 1:**
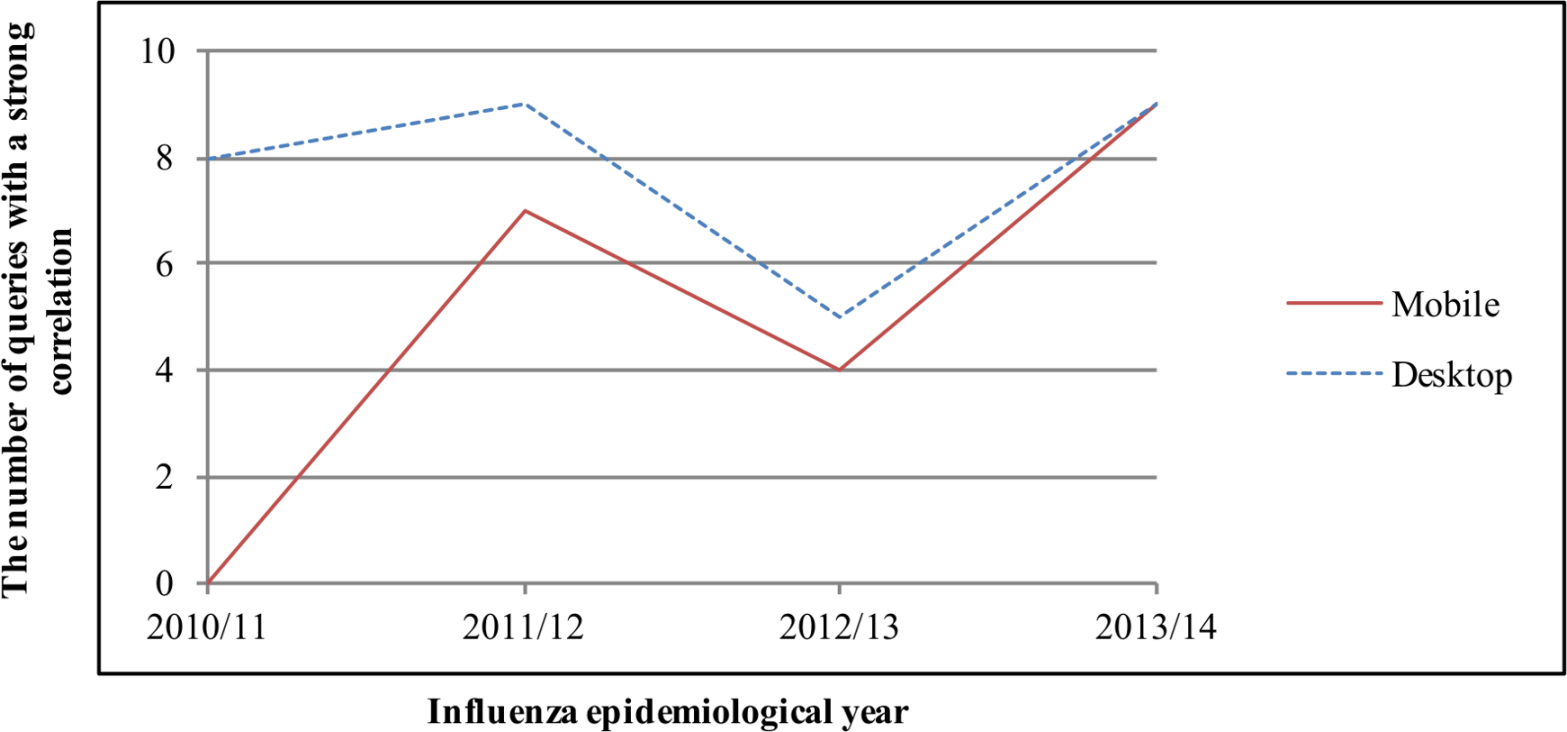
Time series plots of number of search queries having a strong correlation (r-value of ≥ 0.7) with KCDC ILI data.

Among the 210 combined queries, 14 combined queries had statistically significant correlation coefficients with the KCDC data. The coefficients of correlation of 14 queries between the KCDC ILI data and search queries are shown in Table 1. The correlation coefficients of mobile search queries ranged from 0.390 in the 2010/11 epidemiological year ("Bird flu" in Korean: “조류독감”) to 0.910 in 2013/14 ("Bad cold" in Korean: “독감”) and the correlation coefficients of desktop search queries ranged from 0.291 in 2011/12 ("New flu" abbreviation in Korean: “신플”) to 0.931 in 2011/12 ("Tamiflu" in Korean: “타미플루”). The mean coefficients of desktop search queries were higher than those of mobile search queries in 2010/11. However, since 2012/13, the mean coefficients of mobile search queries have been higher than those of desktops (Table 1, Fig 1). No mobile search queries strongly correlated with the KCDC ILI data in the 2010/11 epidemiological year. However, in 2013/14, there were 9 strongly correlated queries for both mobile and desktop searches (Table 1).

**Table 1:** Correlation coefficients between KCDC ILI data and mobile and desktop device search query data from epidemiological years 2010/11 through to 2013/14.

		Mobile search				Desktop search			
Actual search query	English translation	2010/11	2011/12	2012/13	2013/14	2010/11	2011/12	2012/13	2013/14
독감	Bad cold	0.485	0.897	0.778	0.910	0.818	0.863	0.681	0.903
조류독감	Bird flu	0.390	0.504	0.402	0.846	0.903	0.524	0.413	0.842
유행성독감	Epidemiological bad cold	N/A	0.851	0.666	0.805	0.646	0.779	0.518	0.746
플루	Flu	N/A	0.567	N/A	0.700	0.801	0.770	N/A	0.784
H1N1	H1N1†	N/A	0.554	0.571	0.670	0.856	0.630	N/A	0.750
인플루엔자	Influenza	N/A	0.823	0.656	0.880	0.784	0.846	0.731	0.882
Influenza	Influenza (English)†	N/A	0.564	0.638	0.787	0.705	0.838	0.751	0.793
신종독감	New bad cold	N/A	0.784	0.698	0.707	0.382	0.672	0.382	0.510
신종플루	New flu	0.641	0.886	0.788	0.736	0.542	0.802	0.763	0.624
신플	New flu (abbreviation) ‡	N/A	N/A	N/A	0.546	0.574	0.291	N/A	0.326
신종인플루엔자	New influenza	0.443	0.625	0.614	0.664	0.648	0.750	0.538	0.705
돼지독감	Swine flu	N/A	0.539	0.400	0.560	0.757	0.488	N/A	0.628
타미플루	Tamiflu	N/A	0.756	0.846	0.667	0.870	0.931	0.864	0.768
Tamiflu	Tamiflu (English)†	N/A	0.721	0.759	0.734	0.323	0.812	0.740	0.684
Mean coefficient (mean ± SD)		0.490 ± 0.108	0.698 ± 0.144	0.651 ± 0.141	0.729 ± 0.109	0.686 ± 0.179	0.714 ± 0.176	0.638 ± 0.164	0.710 ± 0.153
The number of queries with a strong correlation (r-value ≥ 0.7)		0	7	4	9	8	9	5	9

ILI, influenza-like illness; KCDC, Korea Centers for Disease Control and Prevention; N/A, not applicable due to no Naver data or lack of statistical significance. Naver Trends did not report a value if there were too few searches in a given period; all values are *P* < 0.05 except N/A.

^†^The query was originally submitted in English. All of the other queries were in Korean.

^‡^“New flu (abbreviation) (신플)” is the “New flu (신종플루)” abbreviation in Korean.

### Correlation between the KCDC virologic data and search queries

**Fig 2:**
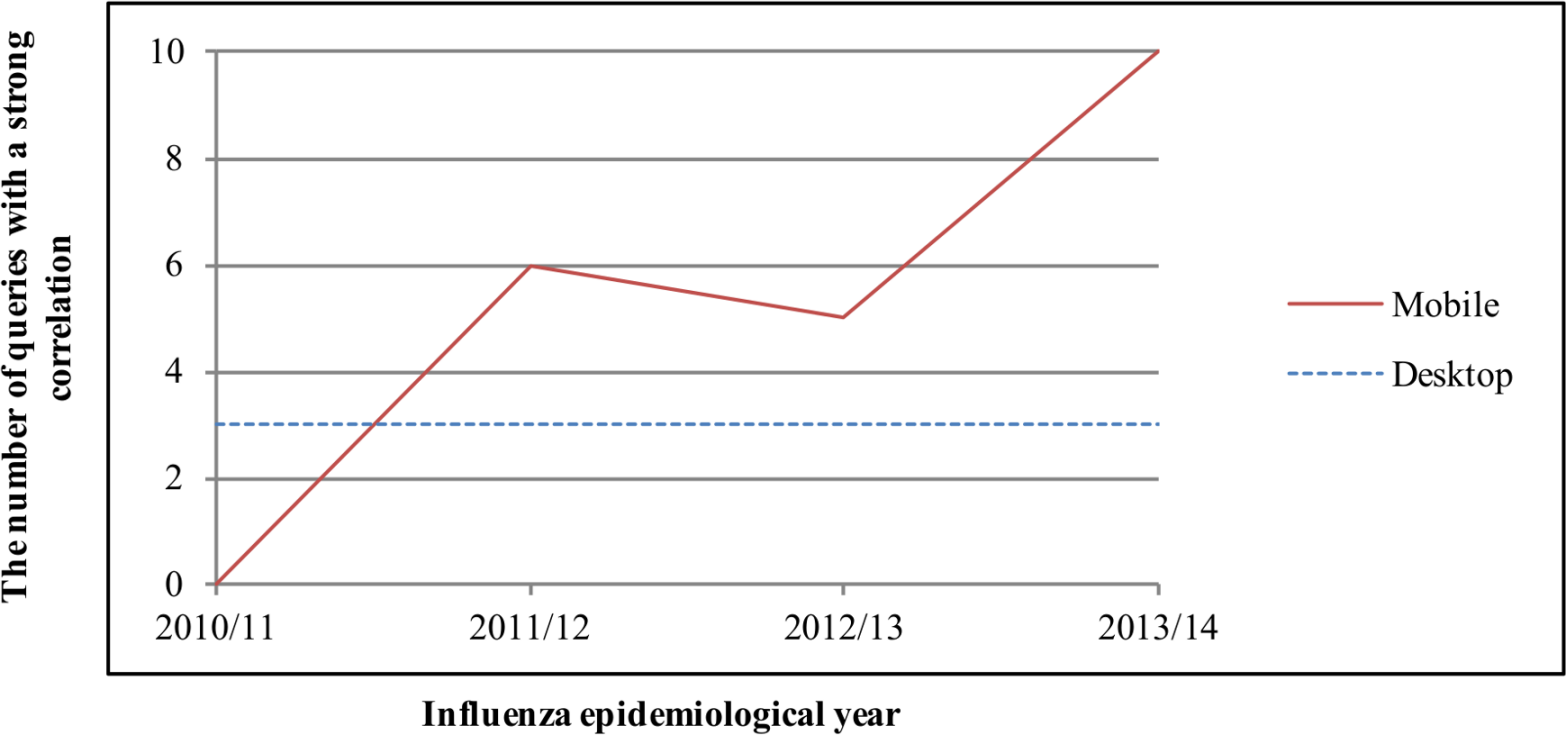
Time series plots of number of search queries having a strong correlation (r-value of ≥ 0.7) with KCDC virologic data.

The coefficients of correlation between KCDC virologic data and search queries are shown in Table 2. The correlation coefficients of mobile search queries ranged from 0.325 in 2010/11 ("Influenza" in Korean: “인플루엔자”) to 0.861 in 2011/12 ("Tamiflu" in Korean: “타미플루”), whereas those of desktop search queries ranged from 0.280 in 2011/12 ("New flu" abbreviation in Korean: “신플”) to 0.841 in 2013/14 ("Tamiflu" in Korean: “타미플루”). The mean coefficients of desktop search queries were higher than those of mobile search queries in 2010/11. However, since 2011/12, the mean coefficients of mobile search queries have been higher than those of desktops (Table 2, Fig 2). No mobile search queries strongly correlated with KCDC virologic data in the 2010/11 epidemiological year but, since 2011/12, mobile search queries have shown better correlations than those of desktops. The correlation trend of the virologic data was similar to that of the ILI data.

**Table 2:** Correlation coefficients^a^ between KCDC virologic data and mobile and desktop device search query data from epidemiological years 2010/11 through to 2013/14.

		Mobile search				Desktop search			
Actual search query	English translation	2010/11	2011/12	2012/13	2013/14	2010/11	2011/12	2012/13	2013/14
독감	Bad cold	0.472	0.722	0.578	0.734	0.553	0.667	0.456	0.626
조류독감	Bird flu	0.601	0.655	0.512	0.736	0.742	0.514	0.516	0.624
유행성독감	Epidemiological bad cold	N/A	0.774	0.632	0.79	0.442	0.673	0.448	0.687
플루	Flu	N/A	0.702	N/A	0.645	0.663	0.805	N/A	0.526
H1N1	H1N1†	N/A	0.486	0.469	0.746	0.710	0.358	0.377	0.633
인플루엔자	Influenza	0.325	0.661	0.722	0.727	0.587	0.629	0.595	0.651
Influenza	Influenza (English)†	N/A	0.584	0.651	0.745	0.687	0.688	0.686	0.747
신종독감	New bad cold	N/A	0.760	0.732	0.717	0.282	0.681	0.395	0.518
신종플루	New flu	0.632	0.782	0.702	0.750	0.421	0.623	0.709	0.650
신플	New flu (abbreviation) ‡	N/A	N/A	N/A	0.559	0.444	0.28	N/A	0.319
신종인플루엔자	New influenza	0.435	0.662	0.677	0.554	0.412	0.541	0.535	0.447
돼지독감	Swine flu	N/A	0.620	N/A	0.672	0.57	0.314	N/A	0.619
타미플루	Tamiflu	0.546	0.861	0.756	0.826	0.719	0.829	0.738	0.841
Tamiflu	Tamiflu (English) †	N/A	0.698	0.731	0.782	0.317	0.797	0.760	0.750
Mean coefficient (mean ± SD)		0.502 ± 0.114	0.690 ± 0.096	0.651 ± 0.095	0.713 ± 0.080	0.539 ± 0.154	0.600 ± 0.178	0.565 ± 0.140	0.617 ± 0.132
The number of queries with a strong correlation (r-value ≥ 0.7)		0	6	5	10	3	3	3	3

ILI, influenza-like illness; KCDC, Korea Centers for Disease Control and Prevention; N/A, not applicable due to no Naver data or lack of statistical significance. Naver Trends did not report a value if there were too few searches in a given period; all values are *P* < 0.05 except N/A.

^†^The query was originally submitted in English. All of the other queries were in Korean.

^‡^“New flu (abbreviation) (신플)” is the “New flu (신종플루)” abbreviation in Korean.

### Lag correlation analysis

The lag correlation analysis of up to two weeks showed similar trends to those seen in Tables 1 and 2. The mean coefficients of mobile search queries and the number of queries with a strong correlation became equal or greater than those of desktop searches over time (S1–S4 Figs and S1–S4 Tables).

## Discussion

The findings of our present study indicate that the performance of mobile search queries for influenza surveillance has equaled or exceeded that of desktop search queries over time. Many people use internet searches to obtain health information before visiting the hospital [9, 36]. Hence, search query trends can reflect actual disease dissemination earlier than conventional surveillance systems. Previous studies have shown that internet search queries highly correlate with conventional influenza surveillance data [8–11]. However, there are some criticisms about digital surveillance, such as Google Flu Trends [20, 21, 23]. The first is that digital surveillance can be used as complementary source but not as a substitute for conventional disease surveillance systems [10, 20–23]. The second is the noises such as changing user behavior, media stoked panic and search engine algorithm change can affect the performance of digital surveillance systems [11, 22, 23].

In the perspective of changing user behavior, we focused on mobile search queries, because the previous studies did not distinguish mobile and desktop searches [8, 10, 11, 14, 17, 25]. It can be important to distinguish between mobile and desktop search queries for several reasons. First, the mobile search volume is rapidly expanding. In this study, we observed that the performance of mobile search queries for influenza surveillance has improved over time. If this change continues in the future, the importance of mobile searches will increase. Second, the correlation coefficients of all mobile queries except N/A in 2010/11 increased in 2013/14. Because 2010/11 was an early phase of the mobile era in South Korea, there may have been too few searches in 2010/11 (N/A in Tables 1 and 2). It is possible that the increase in mobile search volume was also accompanied by a change in search behavior. Several studies have also reported that mobile and desktop user search patterns differ [27–32].

Noise seems to affect both mobile and desktop searches. In Figs 1 and 2, a decrease in the number of search queries having a strong correlation with KCDC ILI and virologic data for both mobile and desktop searches was observed in 2012/13. We do not know exactly why, but search queries are changing [10, 11, 22, 23]. Moreover, other factors, such as media-stoked panic and changes in search algorithms, may have influenced this [10, 11, 22, 23]. However, despite the probable noise, we observed that the influenza surveillance performance of mobile search queries equaled or exceeded that of desktop search queries over time in this study.

Model output, such as Google Flu Trends, showed high correlation coefficients with conventional surveillance systems for influenza. In Europe, correlation coefficients of 0.716 to 0.940 have been reported for Google Flu Trends [8], and coefficients of 0.80 to 0.99 have been reported in the United States [9]. However, individual internet time series, including this study, cannot be compared directly to model output. In our results with KCDC ILI data, the correlation coefficients of mobile and desktop search queries ranged from 0.390 to 0.910 and from 0.291 to 0.931, respectively. These values are similar to or lower than those reported elsewhere [17, 18]. The different queries may have influenced the performance. Queries used prior to this study only reflected the authors’ opinions [17] or were obtained from databases [9, 18]. To obtain population search queries, we used a survey. In our previous study [11], we performed a survey to gather population search queries related to influenza and combined the queries from the results of the survey to reflect people’s search behavior [27].

There were several limitations to this study. First, the queries were obtained in 2012. Compared to our previous study, fewer queries had statistically significant correlations. This may have influenced the performance. However, we thought that it could be appropriate to use only the 14 statistically significant queries to show the change in the performance of mobile queries for influenza surveillance. Second, queries were not collected by separating the mobile and desktop searches. Several studies have reported that mobile and desktop user search patterns differ [27–32], and a similar result was observed in this study. If specific queries on mobile devices are obtained in additional studies, the surveillance performance may be improved. Third, the first day of a KCDC epidemiological week is Sunday. However, the first day of search data from Naver Trends is Monday, and we could not change this. Therefore, it could have skewed the lag analysis. However, we performed the lag analysis using weekly data to minimize the impact. Lastly, we performed this study using open data providing only relative values. Therefore, the results could not be assessed whether the differences were statistically significant, and whether the differences were due to something meaningful or a mechanical artifact.

In summary, we here compared the digital influenza surveillance performances of mobile and desktop search queries. The volume of mobile searches is estimated to surpass desktop searches in the very near future. Our study found that the performance of mobile search queries for influenza surveillance equaled or exceeded that of desktop search queries over time in the study period. In addition, it is possible that the increase in mobile search volume was also accompanied by a change in search behavior. However, we could not show statistically proven difference of correlation or exact causes of these change, since this study was based on limited open data. In the future development of influenza surveillance using search queries, the recognition of changing trend of mobile search data could be necessary.

## Supporting Information

S1 Fig Time series plots of number of search queries having a strong correlation (r-value of ≥ 0.7) with KCDC ILI data from lag correlation analysis (one week preceding of search query). (TIF)

S2 Fig Time series plots of number of search queries having a strong correlation (r-value of ≥ 0.7) with KCDC ILI data from lag correlation analysis (two weeks preceding of search query). (TIF)

S3 Fig Time series plots of number of search queries having a strong correlation (r-value of ≥ 0.7) with KCDC virologic data from lag correlation analysis (one week preceding of search query). (TIF)

S4 Fig Time series plots of number of search queries having a strong correlation (r-value of ≥ 0.7) with KCDC virologic data from lag correlation analysis (two weeks preceding of search query). (TIF)

S1 File All combined queries and raw data of this study. (XLS)

S1 Table Lag correlation analysis (one week preceding of search query) between search query data and KCDC ILI. (DOCX)

S2 Table Lag correlation analysis (two weeks preceding of search query) between search query data and KCDC ILI. (DOCX)

S3 Table Lag correlation analysis (one week preceding of search query) between search query data and KCDC virologic data. (DOCX)

S4 Table Lag correlation analysis (two weeks preceding of search query) between search query data and KCDC virologic data. (DOCX)
