# Supplementary figures and images for: Correlation between National Influenza Surveillance Data and Search Queries from Mobile Devices and Desktops in South Korea
Source: PLoS One. 2016 Jul 8;11(7):e0158539. doi: 10.1371/journal.pone.0158539 (PMC4938422; doi:10.1371/journal.pone.0158539)

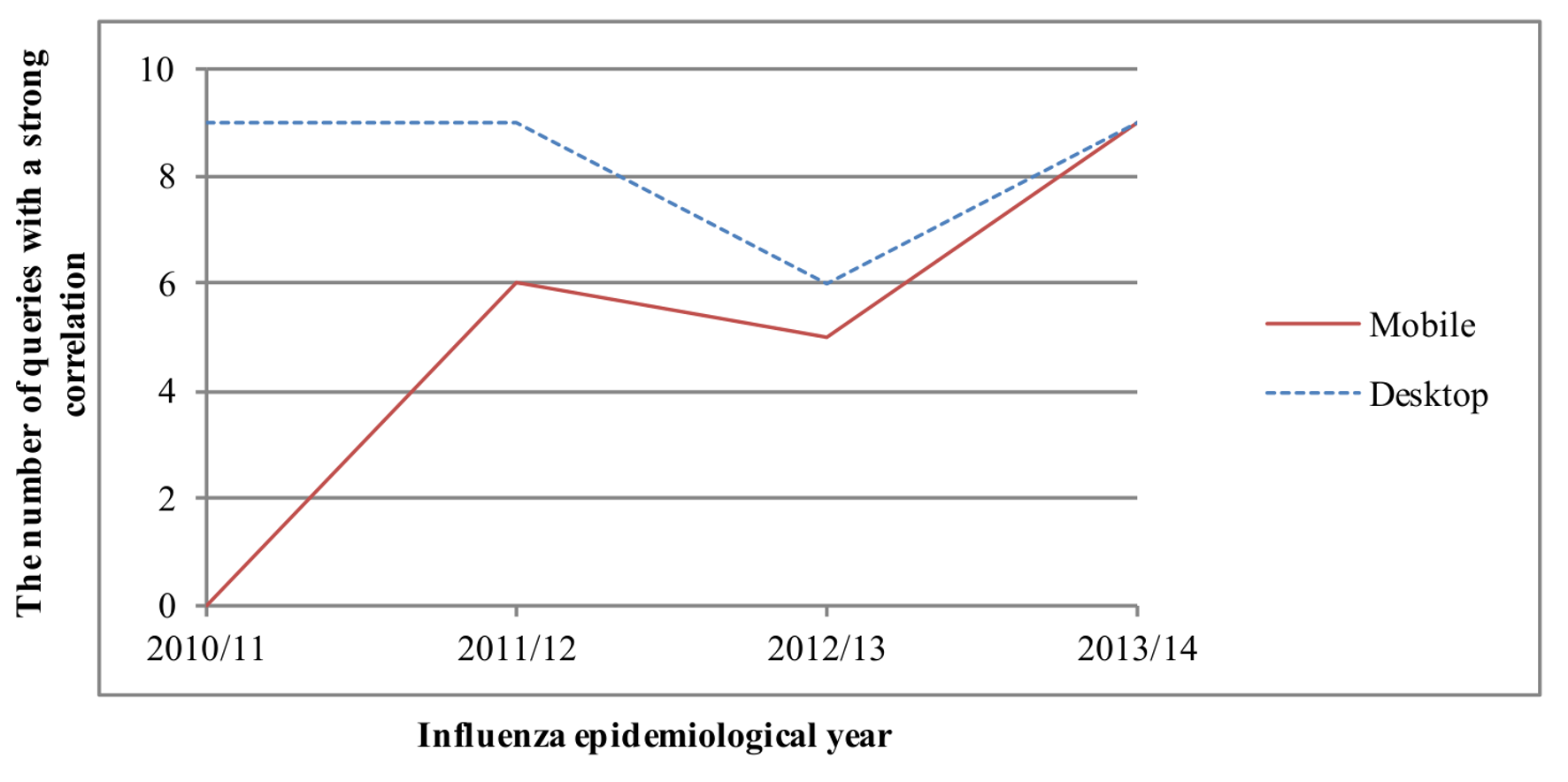

Supplement: S1 Fig — (TIF) [file pone.0158539.s001.tif]

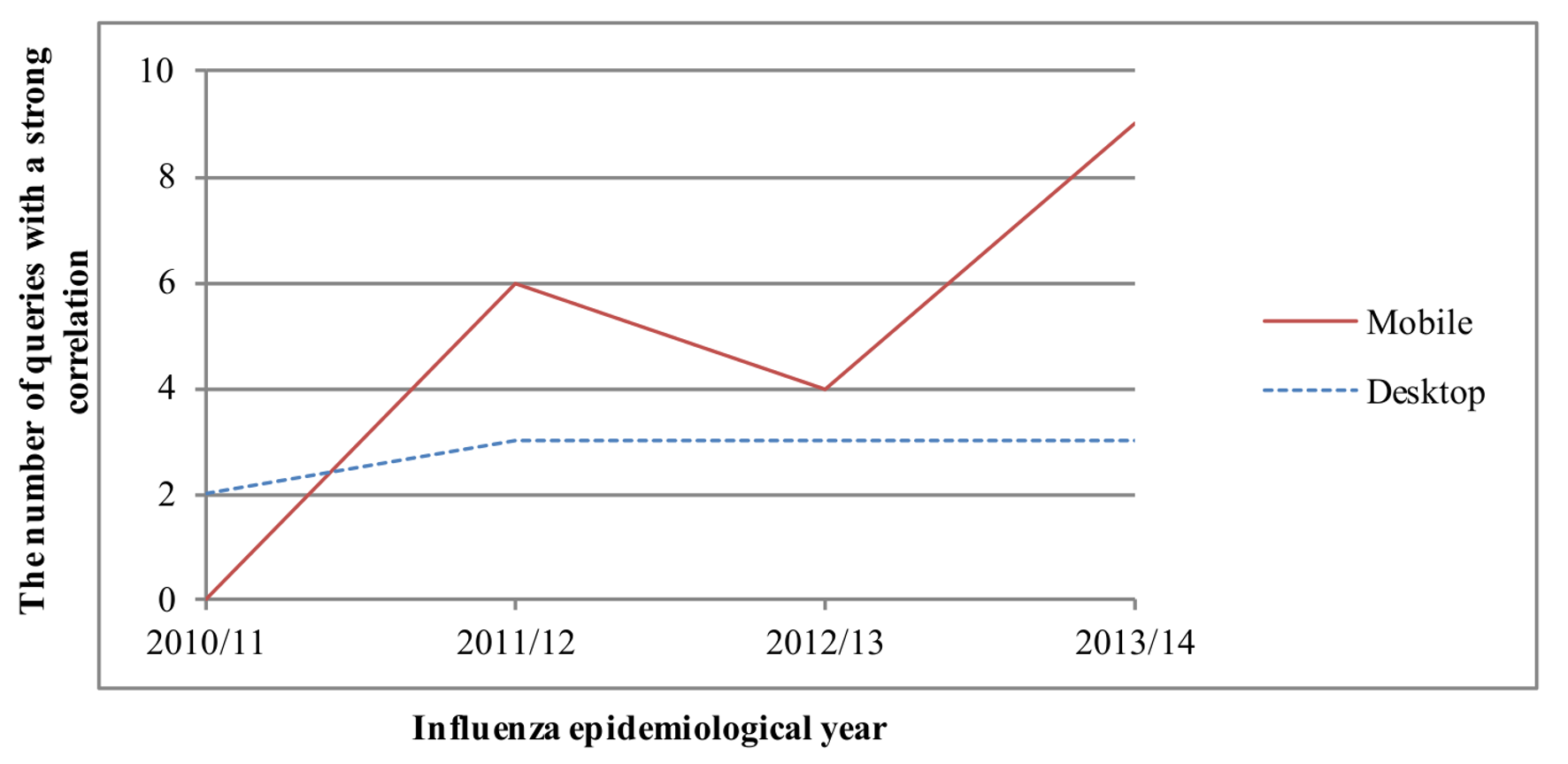

Supplement: S2 Fig — (TIF) [file pone.0158539.s002.tif]

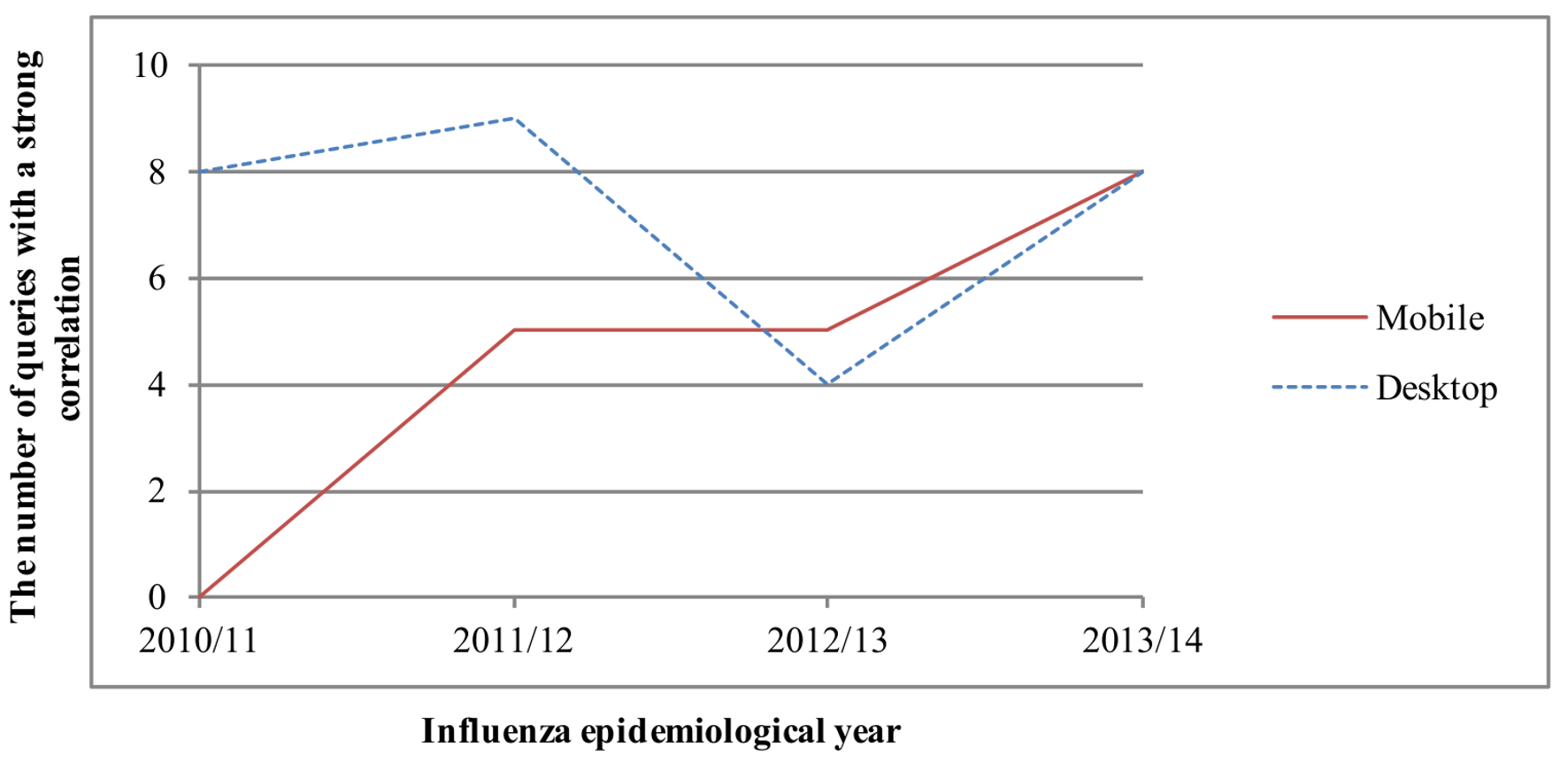

Supplement: S3 Fig — (TIF) [file pone.0158539.s003.tif]

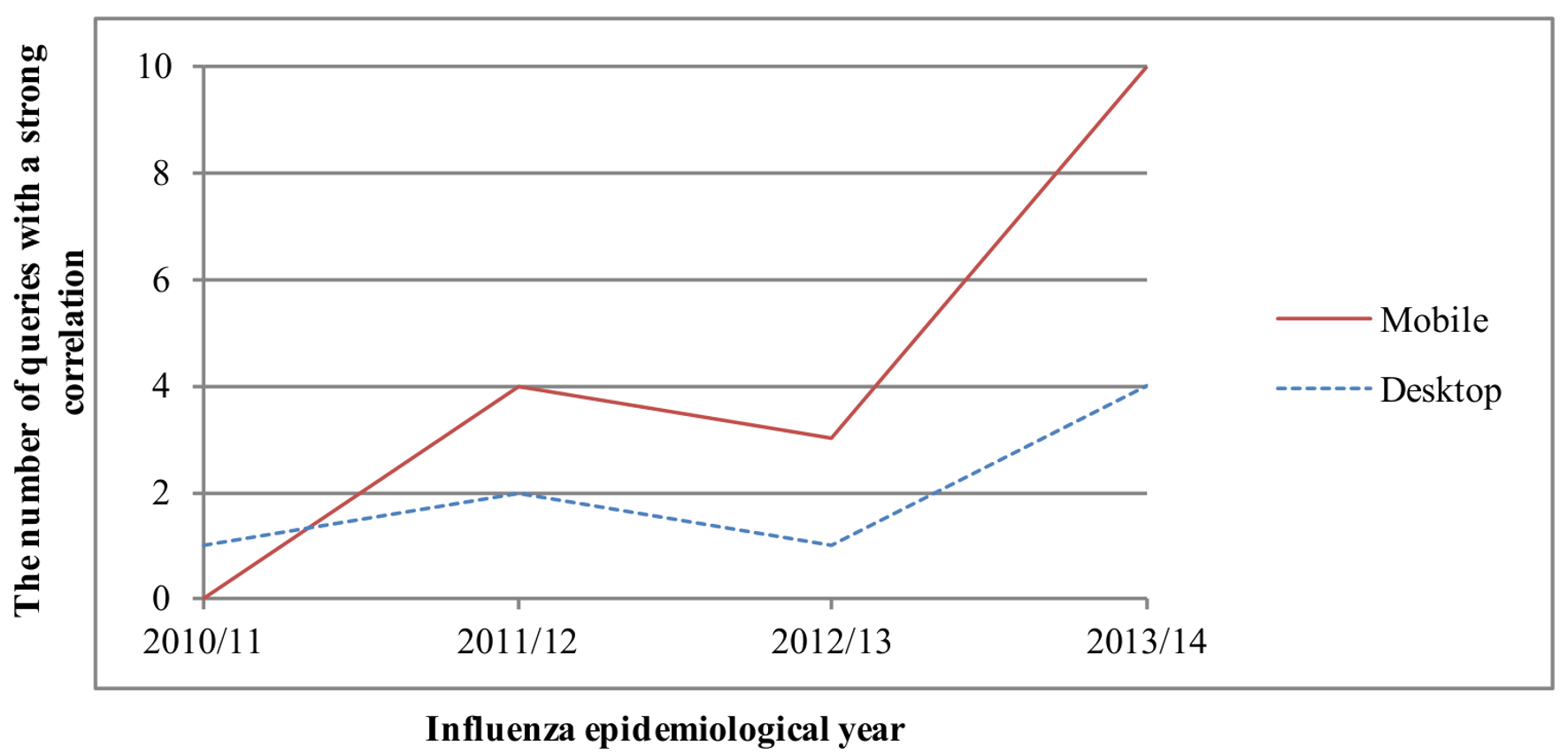

Supplement: S4 Fig — (TIF) [file pone.0158539.s004.tif]
