# Supplementary material for: Correlation between National Influenza Surveillance Data and Search Queries from Mobile Devices and Desktops in South Korea
Source: PLoS One. 2016 Jul 8;11(7):e0158539. doi: 10.1371/journal.pone.0158539 (PMC4938422; doi:10.1371/journal.pone.0158539)
Supplement: S1 Table — (DOCX) [file pone.0158539.s006.docx]

Supplementary Table S1. Lag correlation analysis (1 week preceding) between search query data and KCDC ILI.

|  |  | Mobile search | | | | Desktop search | | | |
| --- | --- | --- | --- | --- | --- | --- | --- | --- | --- |
| Actual search query | Query | 2010/11 | 2011/12 | 2012/13 | 2013/14 | 2010/11 | 2011/12 | 2012/13 | 2013/14 |
| 독감 | Bad cold | 0.563 | 0.876 | 0.824 | 0.902 | 0.881 | 0.857 | 0.723 | 0.913 |
| 조류독감 | Bird flu | 0.310 | 0.552 | 0.354 | 0.843 | 0.864 | 0.599 | 0.361 | 0.865 |
| 유행성독감 | Epidemiological bad cold | N/A | 0.795 | 0.687 | 0.814 | 0.710 | 0.768 | 0.555 | 0.753 |
| 플루 | Flu | N/A | 0.568 | N/A | 0.732 | 0.797 | 0.765 | 0.276 | 0.810 |
| H1N1 | H1N1† | N/A | 0.533 | 0.571 | 0.669 | 0.859 | 0.623 | N/A | 0.766 |
| 인플루엔자 | Influenza | N/A | 0.784 | 0.632 | 0.877 | 0.821 | 0.820 | 0.755 | 0.891 |
| Influenza | Influenza (English)† | N/A | 0.525 | 0.628 | 0.756 | 0.691 | 0.802 | 0.709 | 0.760 |
| 신종독감 | New bad cold | N/A | 0.748 | 0.712 | 0.709 | 0.424 | 0.656 | 0.408 | 0.508 |
| 신종플루 | New flu | 0.693 | 0.922 | 0.808 | 0.731 | 0.566 | 0.852 | 0.762 | 0.607 |
| 신플 | New flu (abbreviation) ‡ | N/A | N/A | N/A | 0.536 | 0.604 | 0.279 | N/A | 0.336 |
| 신종인플루엔자 | New influenza | 0.464 | 0.587 | 0.603 | 0.707 | 0.715 | 0.734 | 0.518 | 0.737 |
| 돼지독감 | Swine flu | N/A | 0.541 | 0.424 | 0.545 | 0.803 | 0.487 | N/A | 0.643 |
| 타미플루 | Tamiflu | N/A | 0.720 | 0.876 | 0.613 | 0.901 | 0.915 | 0.861 | 0.724 |
| Tamiflu | Tamiflu (English)† | N/A | 0.684 | 0.724 | 0.675 | 0.302 | 0.777 | 0.704 | 0.642 |
| Mean of coefficient (mean ± SD) | | 0.508 ± 0.162 | 0.680 ± 0.138 | 0.653 ± 0.155 | 0.722 ± 0.112 | 0.710 ± 0.180 | 0.710 ± 0.170 | 0.603 ± 0.191 | 0.711 ± 0.156 |
| The number of queries with a strong correlation (r-value ≥ 0.7) | | 0 | 6 | 5 | 9 | 9 | 9 | 6 | 9 |

ILI, influenza-like illness; KCDC, Korea Centers for Disease Control and Prevention; N/A, not applicable due to no Naver data or lack of statistical significance. Naver Trends did not report a value if there are too few searches in a given period.; All values of correlation coefficients were *P* < 0.05 except N/A.

^†^The query was originally submitted in English. All of the other queries were in Korean.

^‡^“New flu (abbreviation) (신플)” is the “New flu (신종플루)” abbreviation in Korean.
