# Supplementary material for: Correlation between National Influenza Surveillance Data and Search Queries from Mobile Devices and Desktops in South Korea
Source: PLoS One. 2016 Jul 8;11(7):e0158539. doi: 10.1371/journal.pone.0158539 (PMC4938422; doi:10.1371/journal.pone.0158539)
Supplement: S2 Table — (DOCX) [file pone.0158539.s007.docx]

Supplementary Table S2. Lag correlation analysis (1 week preceding) between search query data and KCDC virologic data.

|  |  | Mobile search | | | | Desktop search | | | |
| --- | --- | --- | --- | --- | --- | --- | --- | --- | --- |
| Actual search query | Query | 2010/11 | 2011/12 | 2012/13 | 2013/14 | 2010/11 | 2011/12 | 2012/13 | 2013/14 |
| 독감 | Bad cold | 0.372 | 0.712 | 0.612 | 0.745 | 0.610 | 0.635 | 0.508 | 0.669 |
| 조류독감 | Bird flu | 0.501 | 0.754 | 0.546 | 0.757 | 0.746 | 0.614 | 0.534 | 0.667 |
| 유행성독감 | Epidemiological bad cold | N/A | 0.757 | 0.677 | 0.809 | 0.402 | 0.671 | 0.496 | 0.723 |
| 플루 | Flu | N/A | 0.745 | N/A | 0.680 | 0.591 | 0.839 | 0.290 | 0.543 |
| H1N1 | H1N1† | N/A | 0.418 | 0.485 | 0.699 | 0.706 | 0.366 | 0.300 | 0.598 |
| 인플루엔자 | Influenza | N/A | 0.631 | 0.682 | 0.715 | 0.569 | 0.591 | 0.628 | 0.682 |
| Influenza | Influenza (English)† | N/A | 0.522 | 0.644 | 0.750 | 0.656 | 0.642 | 0.679 | 0.678 |
| 신종독감 | New bad cold | N/A | 0.695 | 0.735 | 0.721 | N/A | 0.610 | 0.421 | 0.511 |
| 신종플루 | New flu | 0.562 | 0.725 | 0.717 | 0.749 | 0.429 | 0.567 | 0.702 | 0.639 |
| 신플 | New flu (abbreviation) ‡ | N/A | N/A | N/A | 0.542 | 0.390 | N/A | N/A | 0.342 |
| 신종인플루엔자 | New influenza | 0.382 | 0.610 | 0.602 | 0.606 | 0.418 | 0.520 | 0.492 | 0.489 |
| 돼지독감 | Swine flu | N/A | 0.573 | N/A | 0.635 | 0.580 | 0.380 | N/A | 0.667 |
| 타미플루 | Tamiflu | 0.473 | 0.836 | 0.765 | 0.758 | 0.693 | 0.816 | 0.759 | 0.785 |
| Tamiflu | Tamiflu (English)† | N/A | 0.627 | 0.726 | 0.757 | 0.296 | 0.759 | 0.773 | 0.731 |
| Mean of coefficient (mean ± SD) | | 0.458 ± 0.081 | 0.662 ± 0.113 | 0.654 ± 0.086 | 0.709 ± 0.072 | 0.545 ± 0.143 | 0.616 ± 0.143 | 0.549 ± 0.164 | 0.623 ± 0.117 |
| The number of queries with a strong correlation (r-value ≥ 0.7) | | 0 | 6 | 4 | 9 | 2 | 3 | 3 | 3 |

ILI, influenza-like illness; KCDC, Korea Centers for Disease Control and Prevention; N/A, not applicable due to no Naver data or lack of statistical significance. Naver Trends did not report a value if there are too few searches in a given period.; All values of correlation coefficients were *P* < 0.05 except N/A.

^†^The query was originally submitted in English. All of the other queries were in Korean.

^‡^“New flu (abbreviation) (신플)” is the “New flu (신종플루)” abbreviation in Korean.
