# Supplementary material for: Correlation between National Influenza Surveillance Data and Search Queries from Mobile Devices and Desktops in South Korea
Source: PLoS One. 2016 Jul 8;11(7):e0158539. doi: 10.1371/journal.pone.0158539 (PMC4938422; doi:10.1371/journal.pone.0158539)
Supplement: S3 Table — (DOCX) [file pone.0158539.s008.docx]

Supplementary Table S3. Lag correlation analysis (2 week preceding) between search query data and KCDC ILI.

|  |  | Mobile search | | | | Desktop search | | | |
| --- | --- | --- | --- | --- | --- | --- | --- | --- | --- |
| Actual search query | Query | 2010/11 | 2011/12 | 2012/13 | 2013/14 | 2010/11 | 2011/12 | 2012/13 | 2013/14 |
| 독감 | Bad cold | 0.474 | 0.886 | 0.863 | 0.883 | 0.850 | 0.854 | 0.773 | 0.912 |
| 조류독감 | Bird flu | N/A | 0.690 | 0.309 | 0.834 | 0.841 | 0.728 | 0.321 | 0.883 |
| 유행성독감 | Epidemiological bad cold | N/A | 0.759 | 0.698 | 0.807 | 0.677 | 0.772 | 0.592 | 0.757 |
| 플루 | Flu | -0.287 | 0.614 | N/A | 0.735 | 0.769 | 0.780 | 0.314 | 0.805 |
| H1N1 | H1N1† | N/A | 0.493 | 0.507 | 0.652 | 0.852 | 0.602 | N/A | 0.756 |
| 인플루엔자 | Influenza | N/A | 0.771 | 0.583 | 0.868 | 0.807 | 0.812 | 0.740 | 0.890 |
| Influenza | Influenza (English)† | N/A | 0.473 | 0.570 | 0.725 | 0.615 | 0.780 | 0.630 | 0.706 |
| 신종독감 | New bad cold | N/A | 0.697 | 0.717 | 0.691 | 0.358 | 0.607 | 0.428 | 0.490 |
| 신종플루 | New flu | 0.664 | 0.888 | 0.766 | 0.702 | 0.594 | 0.821 | 0.740 | 0.556 |
| 신플 | New flu (abbreviation) ‡ | N/A | N/A | N/A | 0.511 | 0.601 | N/A | N/A | 0.319 |
| 신종인플루엔자 | New influenza | 0.465 | 0.549 | 0.524 | 0.773 | 0.709 | 0.675 | 0.437 | 0.788 |
| 돼지독감 | Swine flu | N/A | 0.521 | 0.351 | 0.548 | 0.810 | 0.504 | N/A | 0.622 |
| 타미플루 | Tamiflu | N/A | 0.721 | 0.822 | 0.568 | 0.858 | 0.876 | 0.813 | 0.682 |
| Tamiflu | Tamiflu (English)† | N/A | 0.635 | 0.704 | 0.601 | 0.282 | 0.724 | 0.665 | 0.591 |
| Mean of coefficient (mean ± SD) | | 0.329 ± 0.421 | 0.669 ± 0.138 | 0.618 ± 0.175 | 0.707 ± 0.120 | 0.687 ± 0.184 | 0.733 ± 0.110 | 0.587 ± 0.183 | 0.697 ± 0.168 |
| The number of queries with a strong correlation (r-value ≥ 0.7) | | 0 | 5 | 5 | 8 | 8 | 9 | 4 | 8 |

ILI, influenza-like illness; KCDC, Korea Centers for Disease Control and Prevention; N/A, not applicable due to no Naver data or lack of statistical significance. Naver Trends did not report a value if there are too few searches in a given period.; All values of correlation coefficients were *P* < 0.05 except N/A.

^†^The query was originally submitted in English. All of the other queries were in Korean.

^‡^“New flu (abbreviation) (신플)” is the “New flu (신종플루)” abbreviation in Korean.
