# Supplementary material for: Correlation between National Influenza Surveillance Data and Search Queries from Mobile Devices and Desktops in South Korea
Source: PLoS One. 2016 Jul 8;11(7):e0158539. doi: 10.1371/journal.pone.0158539 (PMC4938422; doi:10.1371/journal.pone.0158539)
Supplement: S4 Table — (DOCX) [file pone.0158539.s009.docx]

Supplementary Table S4. Lag correlation analysis (2 week preceding) between search query data and KCDC virologic data.

|  |  | Mobile search | | | | Desktop search | | | |
| --- | --- | --- | --- | --- | --- | --- | --- | --- | --- |
| Actual search query | Query | 2010/11 | 2011/12 | 2012/13 | 2013/14 | 2010/11 | 2011/12 | 2012/13 | 2013/14 |
| 독감 | Bad cold | 0.441 | 0.652 | 0.636 | 0.751 | 0.660 | 0.627 | 0.511 | 0.699 |
| 조류독감 | Bird flu | 0.533 | 0.723 | 0.502 | 0.789 | 0.769 | 0.651 | 0.496 | 0.692 |
| 유행성독감 | Epidemiological bad cold | N/A | 0.679 | 0.657 | 0.812 | 0.515 | 0.647 | 0.525 | 0.751 |
| 플루 | Flu | N/A | 0.709 | N/A | 0.702 | 0.652 | 0.814 | 0.337 | 0.661 |
| H1N1 | H1N1† | N/A | 0.413 | 0.512 | 0.696 | 0.697 | 0.447 | N/A | 0.596 |
| 인플루엔자 | Influenza | N/A | 0.544 | 0.621 | 0.775 | 0.593 | 0.547 | 0.601 | 0.711 |
| Influenza | Influenza (English)† | N/A | 0.468 | 0.603 | 0.748 | 0.677 | 0.604 | 0.622 | 0.726 |
| 신종독감 | New bad cold | N/A | 0.651 | 0.741 | 0.709 | 0.187 | 0.554 | 0.432 | 0.502 |
| 신종플루 | New flu | 0.619 | 0.754 | 0.688 | 0.734 | 0.360 | 0.633 | 0.638 | 0.615 |
| 신플 | New flu (abbreviation) ‡ | N/A | N/A | N/A | 0.526 | 0.391 | N/A | N/A | 0.337 |
| 신종인플루엔자 | New influenza | 0.443 | 0.534 | 0.570 | 0.660 | 0.448 | 0.507 | 0.406 | 0.549 |
| 돼지독감 | Swine flu | N/A | 0.501 | 0.343 | 0.678 | 0.607 | 0.341 | N/A | 0.597 |
| 타미플루 | Tamiflu | 0.435 | 0.733 | 0.743 | 0.785 | 0.697 | 0.812 | 0.700 | 0.740 |
| Tamiflu | Tamiflu (English)† | N/A | 0.590 | 0.749 | 0.717 | N/A | 0.678 | 0.744 | 0.694 |
| Mean of coefficient (mean ± SD) | | 0.494 ± 0.081 | 0.612 ± 0.111 | 0.614 ± 0.120 | 0.720 ± 0.071 | 0.558 ± 0.168 | 0.605 ± 0.131 | 0.547 ± 0.127 | 0.634 ± 0.113 |
| The number of queries with a strong correlation (r-value ≥ 0.7) | | 0 | 4 | 3 | 10 | 1 | 2 | 1 | 4 |

ILI, influenza-like illness; KCDC, Korea Centers for Disease Control and Prevention; N/A, not applicable due to no Naver data or lack of statistical significance. Naver Trends did not report a value if there are too few searches in a given period.; All values of correlation coefficients were *P* < 0.05 except N/A.

^†^The query was originally submitted in English. All of the other queries were in Korean.

^‡^“New flu (abbreviation) (신플)” is the “New flu (신종플루)” abbreviation in Korean.
